# Impact of Different Bleaching Methods on Surface Roughness, Microhardness, and Tooth-Restoration Interface of Ormocer- and Methacrylate-based Restorative Systems

**DOI:** 10.4317/jced.62614

**Published:** 2025-04-01

**Authors:** Ali Ihsan Alkhuzaie, Mohamed Elshirbeny Elawsya, Naglaa Rizk Elkholany

**Affiliations:** 1 Postgraduate MSc student, Department of Conservative Dentistry, Faculty of Dentistry, Mansoura University, Mansoura, Egypt; 2 Lecturer, Department of Conservative Dentistry, Faculty of Dentistry, Mansoura University, Mansoura, Egypt; 3 Lecturer, Faculty of Dentistry, Mansoura National University, Gamasa, Egypt; 4 Assistant Professor, Department of Conservative Dentistry, Faculty of Dentistry, Mansoura University, Mansoura, Egypt

## Abstract

**Background:**

The current study assessed the impacts of various in-office bleaching materials (light-activated and chemically-activated) on surface roughness, microhardness, and tooth-restoration interface of two composites restorative systems (ormocer-based and methacrylate-based).

**Material and Methods:**

Sixty specimens were prepared for surface roughness and microhardness (2-mm-thickness, 10-mm-diameter) and classified according to restorative materials (n=30 for each group): group A (ormocer-based group) (Admira fusion, Voco, Cuxhaven, Germany) and group B (methacrylate-based group) (Tetric-N-Ceram, Ivoclar Vivadent, Schaan, Liechtenstein). Each group were subdivided into three subgroups (n=10) according to bleaching agent: subgroup 1 (control group, no bleaching), subgroup 2 (bleached with chemically-activated bleaching agent) (Opalescence Boost, Ultradent, USA), and subgroup 3 (bleached with light-activated bleaching agent) (Philips Zoom, Discus, USA). Eighteen maxillary central incisors teeth were subjected to a tooth-restoration interface evaluation (n=9 for each group) and (n=3 for each subgroup). All specimens were finished, polished, and bleached according to manufacturer’s instruction. A three-dimensional optical profilometer (Wyko, Model NT 1100, Veeco, Tucson, USA) was used to measure surface roughness. The microhardness was assessed using Vickers tester (Model HVS-50, Laizhou Huayin Testing Instrument Co., Ltd. China) and a scanning electron microscope (SEM) (JEOL.JSM.6510LV, Japan) was used to evaluate tooth-restoration interface. The level of statistical significance was determined at *p*<0.05.

**Results:**

For both bleaching agents. There was statistically significant increase of surface roughness for both composite materials after bleaching, and vice versa for microhardness (*p*<0.05), and there was no significant difference between bleaching agents (*p*>0.05). A gap was formed after exposure to bleaching agents compared to control group for both restorative systems.

**Conclusions:**

Both bleaching techniques have bad effects on surface roughness, microhardness, and tooth-restoration interface for both ormocer-based and methacrylate-based restorative systems.

** Key words:**Surface roughness, Microhardness, Tooth-restoration interface, In-office vital bleaching, Ormocer-based composite, Methacarylate-based composite.

## Introduction

A greater tendency towards achieving more aesthetically pleasing and whiter teeth has led to the prevalent utilization of bleaching agents in dentistry, attribuTable to their accessibility and safety. This procedure has long been regarded as the most conservative and economical therapy for enhancing tooth brightness and optimizing appearance. The aesthetics of an existing restoration significantly influence therapeutic success. While the initial color match of a light-polymerized restoration can be determined, prolonged color alterations may arise due to surface staining, microleakage, and wear-related surface modifications. While bleaching is procedurally safe, it may compromise dental materials with significant degrading properties. Peroxide-based treatments facilitate whitening by decomposing their peroxides into unsTable free radicals, which decompose big colored molecules via oxidation or reduction reactions. Consequently, these compounds may potentially influence the sealing capability or surface quality of the restoration (1,2).

The primary and well recognized professional bleaching techniques are in-office bleaching methods. This procedure involves the utilization of a high concentration of hydrogen peroxide (HP), varying from 10% to 40%, which can be activated using light sources, heat, or chemical means. This procedure was proposed to reduce the duration of bleaching exposure and to ensure complete control of the process by a dentist or dental hygienist (3). The supposed benefit of light in bleaching lies in its capacity to heat and activate hydrogen peroxide; various forms of illuminated activation exist, including light-emitting diodes (LEDs), plasma arc lamps, halogen lamps, and the latest innovations in light sources, which are lasers. In-office bleaching employs a higher amount of HP, which can be activated by a light source to accelerate and intensify the bleaching process (4,5).

In chemical bleaching, the bleaching agents comprise (HP) in various quantities and formulations, including carbamide peroxide and sodium perborate. During bleaching, the unstable hydrogen peroxide decomposes due to activation into various forms of free radicals including oxygen free radicals (O.), hydroxyl radicals (HO.), and prehydroxyl radicals (HOO.). These oxygen radicals exhibit high reactivity and lack specificity, potentially causing harm to dental tissues, restorative materials, and the interface between them, which is particularly susceptible to degeneration (6,7).

The necessity for dental materials exhibiting superior aesthetics and durability has prompted the research and development of novel composites. The composition, size, shape, distribution, and content of filler particles are critical for the qualities of the composite. Remarkable advancements accomplished in recent years have enhanced filler technology, augmenting the mechanical and aesthetic features of these materials, leading to the development of contemporary nanohybrid and nanoparticle-containing composites. Nevertheless, minimal modifications were made concerning the organic matrix, and numerous conventional dimethacrylate monomers remain in use (8).

Since its creation by Bowen in 1956, bisphenol A-glycidyl methacrylate, or bis-GMA, has been the main monomer employed in composite formulations. Because of its high viscosity, low molecular weight monomers must be added to the mix in order to get the right viscosity in the finished formulation for clinical usage. The dilution monomers, however, cause the composites’ water sorption and polymerization shrinkage to rise. The cured substance also elutes unreacted monomers, which increases its cytotoxicity to pulp cells. Therefore, novel monomers have been researched with the goal of enhancing the composite restorative materials’ qualities. Recently formulated composites featuring various matrix type, such as ormocer are popular now in restorative dentistry. Ormocer based composite Organically modified ceramics featuring a hybrid molecular structure that integrates the hardness of glass with the characteristics of resin primarily consist of ceramic polysiloxane, exhibiting minimal shrinking (1.25%) in contrast to the organic dimethacrylate monomer matrix observed in other resin composites. Because a more densely crosslinked polymer network is formed, the materials containing ormocer are anticipated to exhibit an increase in degree of conversion and microhardness (9,10).

Despite bleaching procedure seems to be comfortable for patients, it has been reported that it can harm existing dental restorations and also oral and tooth tissues by certain researchers. The potency and acidity of the agents utilized have been connected to the bleaching treatment’s negative effects. Bleaching agents can alter the surface structure, and even the chemical and physical characteristics of the restorative materials and the tooth-restoration interface. The chemical softness resulting from bleaching materials may influence the clinical durability of the composite restoration. Despite the prevalent usage of bleaching agents, researches examining the impact of whitening treatments on the surface characteristics of restorative materials, including different types of composites, represent controversially in the literature. Studies have shown that bleaching treatment can either decrease or increase surface microhardness, while other research indicated no significant change in microhardness (11-13).

Numerous researches have investigated the alterations induced via bleaching in the composite characteristics, a material frequently employed in aesthetic dentistry procedures, including color, surface roughness, microhardness, staining susceptibility, and microleakage (6,14,15). Some studies reported significantly increased in composite surface roughness and other studies documented a substantial decline in surface hardness of bleached composites and the other reported increase microleakage in the enamel margins of restoration after different exposure times to dental bleaching (6,14,15). For such explanations, this *in vitro* study was designed to assess the impact of various types of bleaching materials on surface roughness, microhardness and tooth-restoration interface of ormocer- and methacrylate-based composite restorative systems. The first null hypothesis was that bleaching agents would not affect the surface roughness, microhardness, and tooth-restoration interface. The second null hypothesis was that there would be no significant difference between the effects of both bleaching agents on surface roughness, microhardness, and tooth-restoration interface. The third null hypothesis was that there would be no significant difference between surface roughness, microhardness, and tooth-restoration interface of both composite restorative systems before and after bleaching.

## Material and Methods

Two different composite restorative systems (ormocer-based, methacrylate-based) and two bleaching agents (chemically activated, light activated) were utilized in this study. Brand names, manufacturers, and compositions are presented in ( Table 1).

-Sample size calculation 

The sample size calculation was derived from a prior study employing a comparable design (16). G power program version 3.1.9.7 utilized to calculate a minimum sample size of 9 per subgroup based on an effect size of 1.48, employing a test with two tails, an alpha error = 0.05 and a power = 80.0%, the total calculated sample size was be 9 in each subgroup at least. Therefore, 10 specimens were included in each subgroup.

-Study design

Sixty disk-shape specimens for surface roughness and microhardness were prepared and classified according to restorative materials (n=30 for each group): group A (ormocer-based group) and group B (methacrylate-based group). Each group was subdivided into three subgroups (n=10) according to bleaching agent: subgroup 1 (control group, no bleaching), subgroup 2 (bleached with chemically-activated bleaching agent) and subgroup 3 (bleached with light-activated bleaching agent). Eighteen maxillary central incisors teeth subjected to a tooth-restoration interface evaluation. Teeth were classified in the same manner of surface roughness and microhardness (n=9 for each group) and (n=3 for each subgroup).

-Specimen’s preparation for surface roughness and microhardness

For surface roughness and microhardness tests, sixty samples were prepared by using specially designed acrylic mold with dimensions 10mm in diameter and 2mm in thickness. The color for both composite materials that are relevant to shade A2. The acrylic mold was placed on a transparent Mylar strip positioned on a glass slide. The composite materials were inserted in one increment until the mold was slightly over filled. Then, a transparent Mylar strip was set above them and another glass slide was secured in order to flatten the surface. Each sample was subjected to light curing for 40 s from top surfaces, using a LED curing device (Eighteeth, Curingpen-E, Changzhou Sifary Medical Technology Co., Ltd., China), at a right angle and intimate contact to the glass slide at intensity which was approximately 1200 mW/cm2. The intensity was regularly measured with curing radiometer (Bluephase Meter II, Ivoclar Vivadent, Liechtenstein) after each sample. Finishing and polishing were accomplished with aluminum oxide discs from the finishing/polishing system (Tor V M, Russia) according manufacturing instructions utilizing from coarse to fine discs gradually on a low-speed handpiece (Sirona T3, Benshein, germany), at speed 12,000 rpm. All samples were preserved in distilled water at 37 ± 1°C in an incubator (BTC, Model: BT1020, Cairo, Egypt) for 24 h prior to the testing methods.

-Teeth selection and ethical approval

Teeth were gathered from the Oral Surgery Department Clinic at the Faculty of Dentistry, Mansoura University, extracted as a result of periodontal disease. This strategy was carried out in accordance with the standards established by the Faculty of Dentistry Ethical Committee at Mansoura University, under protocol number A0302024CD. After the manual removal of soft tissue remains with a hand scaler (Goldman, Illinois, USA), the teeth were submerged in a 1% Chloramine-T solution for 72 h to disinfect the collected specimens. Eighteen specimens were chosen; all were devoid of cracks as verified through examination at 30x magnification utilizing a binocular stereomicroscope (SZ TP, Olympus, Tokyo, Japan). A rubber cup and a fine pumice water slurry were subsequently employed to clean the teeth. Following that, the teeth were kept in distilled water at 37 ± 1°C in an incubator (BTC, Model: BT1020, Cairo, Egypt).

-Specimen’s preparation for tooth-restoration interface

• Mounting

Each tooth’s crown was removed from its roots 1mm below CEJ with a Low-speed diamond disc (Isomet, Buehler, USA) utilized with water irrigation, and the roots were eliminated. The fabricated crowns were individually positioned in self-curing acrylic resin (Acrostone, Egypt) contained within a cylinder measuring 2 cm in internal diameter and 2 cm in height to facilitate proper handling and to exclude any external contamination during the application of bleaching agents. Upon reaching the dough stage, each specimen was positioned at the center of the cylinder within the acrylic resin mixture. Finally, wet 600, 800, 1000-grit silicon carbide abrasive paper (Microcut M, Buehler, USA) was used to flatten the labial enamel surfaces of teeth. For assurance of surface cleanliness, the teeth were polished with preventive paste (PSP Dental Company, UK, Kent) utilizing a rubber cup and rinsed with distilled water.

• Cavity preparation

Cavity was prepared with dimensions 4mm in diameter and 2mm in depth cylindrical shape in the middle third of labial surface by #009 fissure diamond points (Komet, Germany) with high-speed handpiece (Sirona T3, Benshein, germany). The prepared cavity dimensions were measured by using periodontal probe.

• Restorative procedures

For ormocer-based group, 36% phosphoric acid etchant gel (Vococid, Voco, Cuxhaven, Germany) was administered for 30 s to the enamel surface and 15 s to the dentin surface. After that, it was rinsed with water spray for 20 s. To prevent drying out, any leftover water was blown away with a mild air stream. A layer of Admira Bond (Voco, Cuxhaven, Germany) was employed and rubbed for 30 s and light-cured for 20 s. Then the cavities were filled with Admira Fusion (Voco, Cuxhaven, Germany) composite with one increment of 2 mm and cured for 40 s. For methacrylate-based group, 37% phosphoric acid etchant gel (N-Etch, Ivoclar Vivadent, Schaan, Liechtenstein) was employed for 30 s on the enamel surface and 15 s on the dentin surface. Subsequently, it was subjected to a 20 s water spray, and any excess moisture was removed using a mild air stream. A layer of Tetric-N-Bond (Ivoclar Vivadent, Schaan, Liechtenstein) was applied and rubbed for 20 s and cured for 20 s. Then the cavities were restored with Tetric-N-Ceram (Ivoclar Vivadent, Schaan, Liechtenstein) composite with one increment of 2 mm and cured for 40 s. Finishing and polishing were performed as the same for disks and specimens were preserved in distilled water at 37 ± 1°C in an incubator (BTC, Model: BT1020, Egypt) for 24 h prior the test.

-Bleaching procedures

• Light-activated bleaching agent

Zoom whitening and the activator gels (Discus, Dental, LLC, Ontario CA91761, USA) were combined using an integrated mixing nozzle after being dispensed from the syringe. The 25% HP whitening gel was applied to the specimens using a brush, creating a layer of 1 mm thickness based on the manufacturer’s instructions. The gel was triggered with the Philips Zoom advanced power unit (ZOOM! White Speed Power Pack, Whitening Light Emitting Diode Accelerator, Philips violet wavelength 350-400 nm, and light intensity of 195 mW/cm²), which was calibrated to direct light towards each specimen for 15 min per session. Three sessions were held. The gel was pulled out after each session with a suction tip, followed by washing the specimens with an air/water syringe and drying them with gauze after the final session.

• Chemically-activated bleaching agent 

The Opalescence Boost whitening system was employed, whereby the two syringes were connected, and the little clear plunger of the bleaching agent was squeezed into the central clear syringe to rupture the internal diaphragm, therefore combining the bleaching agent with the activator. The mixture was inverted a minimum of 25 times to ensure complete the combination. Transfer all combined gel into the red syringe, then twist to detach both syringes, after which the required microtip should be fixed to the red syringe. A 40% hydrogen peroxide bleaching gel was administered to the specimens in a 1 mm thick layer, following the manufacturer’s instructions, and left for 15 min for each session. Three sessions were conducted. The gel was pulled out after each session with a suction tip, followed by washing the specimens with an air/water syringe and drying them with gauze after the final session.

-Surface roughness test

**Figure 1:**
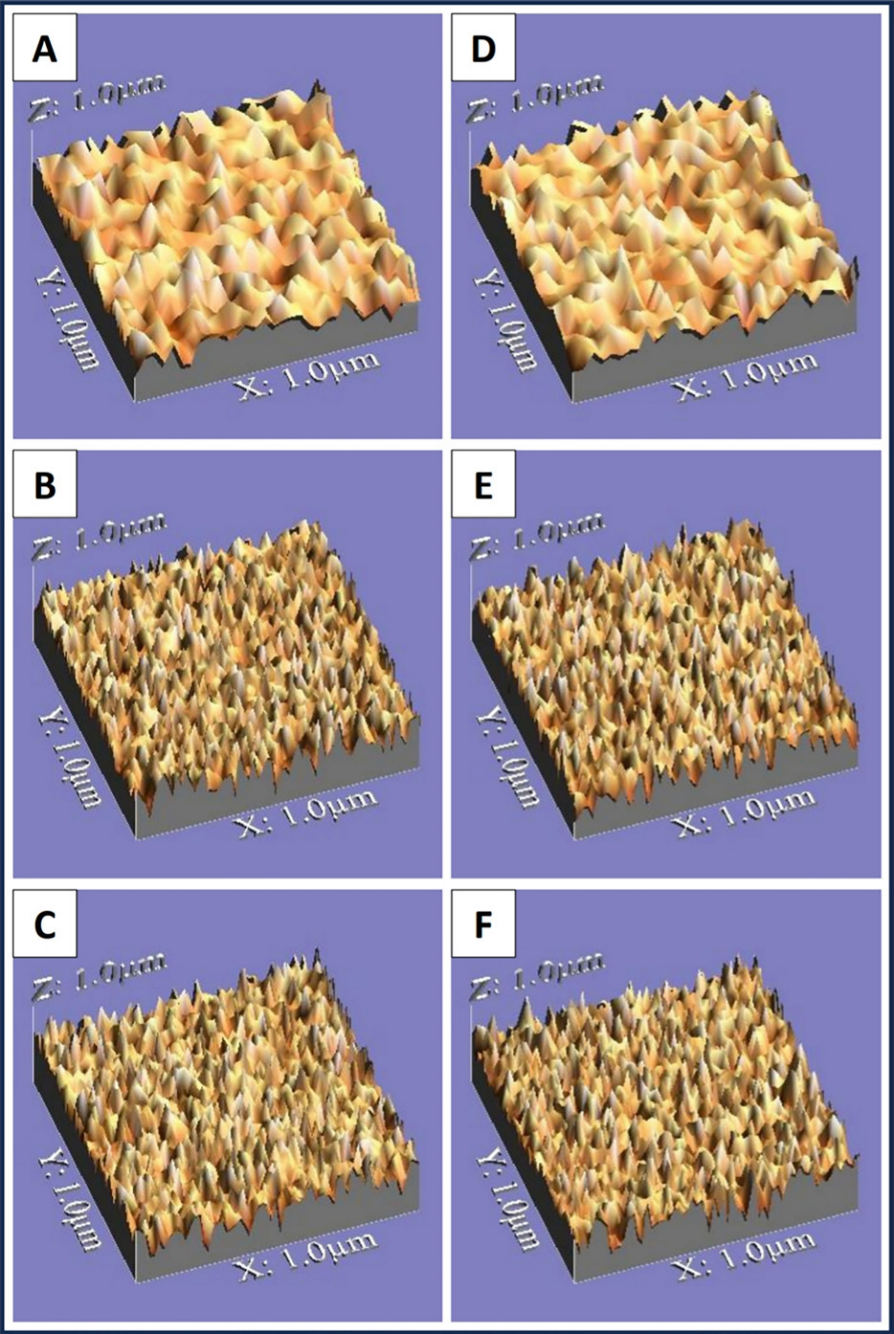
Surface topography of representative specimens from all tested groups. (A), (B), and (C) are for methacrylate-based composite. (D), (E), and (F) are for ormocer-based composite. (A) and (D): for control groups (no bleaching), (B) and (E): for light activated bleaching, (C) and (F): for chemical activated bleaching.

A noncontact three-dimintional optical profilometer (Wyko, Model NT 1100, Veeco, Tucson, USA) connected to a computer with imaging software (Vision 32, Veeco, USA) was utilized to assess surface roughness (17). The program utilized for picture generation provided arithmetic roughness mean (Ra) data derived from the peaks and valleys observed in the investigated region, employing a profilometer with a 0.8 mm cutoff and a 2.4 mm evaluation length. Consequently, a three-dimensional representation of the specimen’s surface profile was generated. Subsequently, five three-dimintional pictures were collected for each specimen in both the central and lateral regions (18). Surface topography of representative specimens from all tested groups are shown in (Fig. 1).

-Microhardness test

Surface microhardness was assessed utilizing a Digital Display Vickers Micro-hardness Tester (Model HVS-50, Laizhou Huayin Testing Instrument Co., Ltd., China) equipped with a Vickers diamond indenter and a 20X objective lens. A load of 100 g was exerted for 15 s. Three indentations, evenly distributed around a circle and separated by a minimum distance of 0.5 mm from neighboring indentations, were created on the surface of each specimen. The lengths of the diagonals of the indentations were determined utilizing a built-in scaled microscope. Microhardness was determined using the subsequent formula: HV=1.854 P/d2 where, HV is Vickers hardness in Kgf/mm2, P is the load in Kgf and d is the length of the diagonals in mm.

-Tooth-restoration interface evaluation

The assessment of the tooth-restoration interface specimens was performed utilizing a scanning electron microscope (JEOL.JSM.6510LV, Japan) at a magnification of x1000. Each specimen was air-dried, fitted to a copper stub, and coated with a thin coating of gold using a sputter evaporator (SPI-Sputter Coater, USA) prior to examination with a scanning electron microscope to assess the margin as either gap-free (intact) or gapped.

-Statistical analysis

The data were analyzed with SPSS® software version 25 (SPSS Inc., Chicago, IL, USA). Shapiro-Wilk tests were employed to assess the normality of the data distribution for all variables. The data were parametric and met the normal distribution. Consequently, descriptive statistics were presented using mean, and standard deviation. Comparison for hardness, and roughness between composite groups (Admira, and Tetric composites) and bleaching groups (control, light, and chemical) was made using two-way ANOVA followed by Bonferroni test for multiple comparisons. *p-value*s <0.05 were regarded as significant.

## Results

-Surface roughness

Two-way ANOVA revealed that there was no significant difference in overall roughness between composite groups (*p*=.722). Even so, there was a significant difference in overall roughness between bleaching techniques (*p*=.012). Also, the interaction composite type*bleaching technique was not significant (*p*=.335).

Comparison of roughness between types of composite for each bleaching technique is presented in ( Table 2). For both bleaching techniques, there was no significant difference in roughness between types of composite (*p*=.891 for control, *p*=.201 for light, and *p*=.420 for chemical).

Comparison of roughness between types of bleaching technique for each composite group is presented in ( Table 2). For both composite groups, there was a significant difference in roughness between bleaching techniques (*p*=.027 for Admira, *p*=.024 for Tetric). The lowest roughness was observed with control, followed by light, and the highest was noted with chemical. Multiple comparison of roughness between bleaching techniques is presented in ( Table 2). For both composite groups, there was a significant difference between control and bleached groups. However, there was no significant difference between light and chemical bleaching techniques.

Vicker’s microhardness

Two-way ANOVA indicated that there was a significant difference in overall hardness between composite groups (*p*<.001) and bleaching techniques (*p*=.005). However, the interaction between composite type*bleaching technique was not significant (*p*=.994).

Comparison of hardness between types of composite for each bleaching technique is presented in ( Table 3). For both bleaching techniques, there was a significant difference in hardness between types of composite (*p*=.009 for control, *p*=.010 for light, and *p*=.013 for chemical). Tetric recorded significantly higher hardness than Admira.

Comparison of hardness between types of bleaching technique for each composite group is presented in ( Table 3). For both composite groups, there was a significant difference in hardness between bleaching techniques (*p*=.049 for Admira, *p*=.045 for Tetric). The greatest hardness was recorded with control, succeeded by light, and the least was noticed with chemical. Multiple comparison of hardness between bleaching techniques are presented in ( Table 3). For both composite groups, there was a significant difference between control and bleached groups. However, there was no significant difference between light and chemical bleaching techniques.

Tooth-restoration interface evaluation

**Figure 2:**
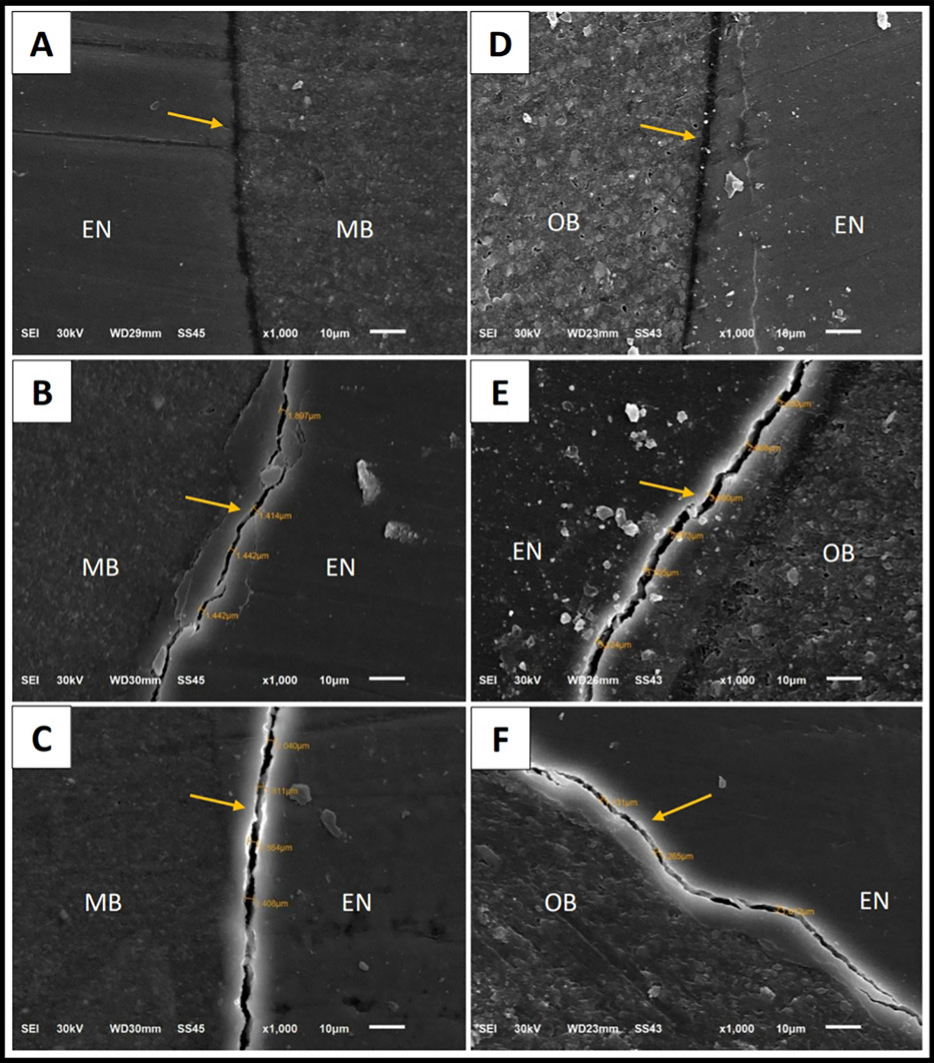
Representative SEM micrographs of the tooth-restoration interface at ×1000 magnification. (A), (B), and (C) are for methacrylate-based composite. (D), (E), and (F) are for ormocer-based composite. (A) and (D): for control groups (no bleaching), (B) and (E): for light activated bleaching, (C) and (F): for chemical activated bleaching. Arrows are directed to tooth-restoration interface showing intact margins for control groups (A and D), and marginal gap for all bleached groups (B, E, C, and F). EN: enamel, MB: methacrylate-based composite, OB: ormocer-based composite.

The tooth-restoration interface was gap free with control group, and there was a gap formed after exposure to light-activated and chemically-activated bleaching agents for two types of composites as shown in (Fig. 2).

## Discussion

Bleaching chemicals brighten stained dental structures by decomposing peroxide into free radicals. Free radicals deconstruct large pigmented molecules, which reflect specific wavelengths of light and contribute to enamel discoloration, into smaller, less pigmented molecules by oxidation and reduction processes. Bleaching agents can lead to a softening and decrease in the microhardness of composite restorative materials, while free radicals generated by peroxides may impact the resin-filler interface, leading to in filler-matrix detachment, which leads to an elevation in surface roughness (6,7). The variations in the roughness and microhardness values of the composites, following the identical bleaching protocol, may be ascribed to the specific polymers inside their organic matrix, along with the quantity and particle size of the fillers. In general, the mechanical and physical characteristics of composite restorative materials are largely dependent on the fillers content, type, size, loading level, and morphology; as well as the matrix content (19).

The whitening of teeth has gained great popularity, the impact of bleaching on esthetic appearance, physical and mechanical properties of dental materials must be taken into account. This complicates the process for attempting to create and sustain strength, glossy surface and adapted margin between the dental restoration and the adjacent tooth structure. As a result, the stain accumulation and gap formation that found in composite restoration, which led to secondary caries and loss of restoration (15). Therefore, this study was performed in order to assess the impact of chemical- and light-activated bleaching agents on physical properties (surface roughness, microhardness) and the marginal integrity of different type of composites (ormocer-based and methacrylate-based).

Admira Fusion is an ormocer composite that enhances aesthetics appeal, biocompatibility, resistance to abrasion, caries protection, and minimizes polymerization shrinkage and surface texture. It is an organically modified ceramic, composed of an inorganic silicon dioxide base and polymerizable organic components; it merges the toughness of glass with the characteristics of resin. This tooth-colored substance aims to enhance aesthetics appearance and resistance to abrasion, reduce polymerization shrinkage and surface texture, and provide protection against caries (9,10). Tetric N-Ceram is a methacrylate-based composite, have clinical evaluation with long-term investigations demonstrated favorable clinical performance across all measures. Tetric-N-Ceram is a nano-hybrid composite composed of ceramic/glass fillers and nano additives that enhance the viscosity and wettability of the filler particles with the resin. Moreover, the manufacturer asserts that the resin utilized in this product has greater hydrolytic stability in acidic and alkaline conditions (19).

This study employs bleaching chemicals with distinct activation modes: light-activated and chemically-activated. The high doses of hydrogen peroxide are designated solely for in-office use. The manufacturer specifies an identical duration for the bleaching session of these products, so precluding any potential influence of bleaching time on our assessment (5). The objective of employing light-activated bleaching chemicals in this study is that their bleaching mechanism dependent on photo-fenton chemistry. This utilizes ferrous gluconate. This reaction produces extremely reactive hydroxyl radicals that are beneficial for bleaching. Nevertheless, the activity of free radicals reduces chair-side time, thereby diminishing the duration of demineralization and its impact on surface roughness and microhardness (4,5). A light-activated bleaching agent (Philips ZOOM), used with active ingredient is 25% HP, is regarded as a cost-efficient substitute for lasers and halogens, as it necessitates reduced energy for light generation with diminished heat output. The blue light emitted by Zoom! does not generate heat. Light functions by cleaving the conjugated double carbon link C=C into C-C single-bonded molecules, hence enhancing the photochemical reaction rate. This technology enhances tooth whitening by ultraviolet light, hence increasing the efficacy of bleaching treatments and subsequently reducing chairside duration (20). Furthermore, the purpose of using Opalescence Boost is a chemically activated in-office whitening gel that provides brighter, whiter teeth after approximately one hour of use. This study includes it because of its popularity as an in-office bleaching method and its recognition as the successful gold-standard tooth whitening procedure utilized in most studies. The potent 40% HP gel is chemically activated, eliminating the need for whitening illuminations in Opalescence teeth whitening, as such lights do not influence the whitening outcomes. Opalescence Boost in-office whitening depends exclusively on chemical activation (21).

This study utilized a non-contact profilometer to assess surface roughness. The utilization of this instrument facilitated measurement of both control and treated specimens, unlike contact profilometers, which induce surface changes and indentations on composite surfaces, potentially leading to inaccurate postoperative results. This study assessed surface roughness due to its significance in biofilm formation and bacterial adhesion, which can contribute to gingival irritation and caries. In dentistry, surface roughness assessments are typically conducted using a profilometer. The arithmetic average roughness values are the most often utilized parameter in evaluating surface roughness (17). The present study utilized an optical laser profilometer to offer a non-contact, nondestructive, and rapid quantitative assessment of surface roughness (18).

The microhardness test was employed because hardness denotes the capacity of restorative composite materials to tolerate mechanical deterioration during use. Hardness is expressed as a material’s resistance to indentation or penetration. This study examined surface microhardness using the Vickers system, which has been employed in several investigations to assess surface alterations due to its accuracy, availability, and simplicity. The Vickers indenter is superior than the Knoop indenter because to its requirement for a consistent square shape, and the minor elongation of the diagonal indentations, which introduces mistakes in hardness measurements, is readily identifiable. It is recommended that the Vickers indenture be consistently employed in hardness studies. Various methods are employed to assess surface hardness, and the selection of the method should depend on the substance being examined. The Vickers test, appropriate for assessing the hardness of brittle materials, can be employed to evaluate the microhardness of composite specimens (16,22).

In the current study, tooth-restoration interface was evaluated as the degradation of the composite surface may lead to appearance of gaps in the tooth-restoration interface and thereby affect the marginal sealing of restorations. Marginal adaption refers to the interfaces between the restoration and the dental structure. Inadequate marginal adaptation may lead to postoperative discomfort, recurrent decay, pulp irritation, and marginal discoloration. The integrity and durability of materials rely on their marginal adaptation to cavity walls, which affects the clinical performance and lifetime of oral restorations. An inadequate marginal seal causes microleakage at the tooth restoration contact, leading to restoration failure (23). SEM was employed for a detailed analysis of the restoration margins due to its capacity for magnification and detail revelation (24).

Result in this study of composite surface roughness evaluation showed significant increase for all tested material between control and bleached group, there was no significant variation in roughness between bleaching techniques. Materials containing resin soften and abrade their surfaces upon exposure to acidic substances, hence increasing their susceptibility to physical pressures. HP is a potent oxidizing agent and highly acidic (3). In the process of HP decomposition, it breaks down into hydroxyl radicals or water and oxygen molecules. HP and liberated free radicals may interact with the organic polymer matrix of the composites and the inorganic structures, ultimately leading to surface dissolution by the extraction of mineral elements (11). This result is in line with Yu *et al*. (25). who analyzed the effects of bleaching on the surface roughness, and found that the roughness of the tested groups increased after bleaching. Also, the result of the current study agrees with Hafez *et al*. (26). who determined surface roughness of composites following in-office bleaching, and found significant increase of surface roughness after bleaching. While this result disagrees with Wattanapayungkul *et al*. (27). who assessed the impact of various types of bleaching concentration were applied to the surface finish of several tooth-colored restorative materials, demonstrating no significant alteration in surface roughness between the bleached and control groups. The observed inconsistencies may be partially attributed to variations in experimental methodology and the bleaching chemicals employed. Furthermore, the result of the current study disagreement with Dogan *et al*. (28). who investigated the effect of three bleaching agents on roughness of three dental composites, and found the roughness of all bleached specimens was greatly reduced in contrast to that of unbleached specimens. May be due to the roughness quantities have been evaluated using an atomic force microscope and a metallographic microscope.

The result of the microhardness showed that for both groups significant decrease in microhardness; the greater hardness was recorded with control, succeeded by light, and the least hardness was noticed with chemical. This was likely attribuTable to the bleaching agent’s softening effect on the matrix of both composites, hence diminishing their surface characteristics (29). These changes revealed to the significant discrepancies in results indicate that certain composite restorative materials may be more prone to modifications, while specific bleaching treatments may induce such changes. The latter may be related to the variations in the power of hydrogen (pH) across bleaching agents, whereas light curing units promote hydrogen peroxide decomposition, hence speeding up chemical processes during the bleaching process. The free radicals generated by the decomposition of HP impact restorative materials. HP possesses a significant oxidizing potential that can influence both the pigment macromolecules and the resin matrix. It also promotes the oxidative degradation of polymer chains in peroxides, resulting in bond failure between the organic and inorganic component (6). The results of the current study are in agreement with Alayad *et al*. (12). who examined the effects of various bleaching regimens by the evaluation of microhardness of composite materials, and found that there was significant decline in the values of microhardness was marked between the control and in-office chemical activated bleaching (opalescence boost 40% HP). Also, the result of the current study is in line with Bahari *et al*. (29). who investigated the effects of different bleaching strategies on the microhardness of dental composite and found that, all the bleaching agents significantly decreased microhardness compared to the control group, the greater mean microhardness in the control group, and the mean values of microhardness in the light-activated 35% HP was significantly higher than chemical-activated. While the result of the current study is in disagreement with Yap *et al*. (30). who investigated the effects of in-office tooth whiteners on the hardness of hybrid composites, and found no substantial variation in hardness was detected between the control and bleached groups. This may be due to different type of composites was used. Also, the result disagrees with Leal *et al*. (22). who evaluated the microhardness of composites material after two bleaching regimens, who found that the microhardness values of methacrylate composite resin surfaces were increased after the application with 35% HP.

The first null hypothesis, which assumed that bleaching agents would not affect the surface roughness, microhardness, and tooth-restoration interface, has been rejected. The second null hypothesis, which assumed that there would be no significant difference between the effects of both bleaching agents on surface roughness, microhardness, and tooth-restoration interface has been accepted. The third null hypothesis, which assumed that there would be no significant difference between surface roughness, microhardness, and tooth-restoration interface of both composite restorative systems before and after bleaching has been largely rejected. Because this *in vitro* study was unable to accurately replicate the intraoral environment, which included dynamic and complex biological variables like saliva, plaque bacterial biofilm, and natural remineralization, direct extrapolations to clinical circumstances should be used with caution. Also, need more composite groups, bleaching agents and thermocycling for further studies.

## Conclusions

Within the limitations of this study we can conclude that, both bleaching techniques (light-activated and chemical-activated) have bad effects on surface roughness, microhardness, and tooth-restoration interface for both composite restorative systems (ormocer-based and methacrylate-based).

## Figures and Tables

**Table 1:** Materials used in this study.

Material	Composition	Filler size	Filler content	Manufacturer	Batch Number
Tetric N Ceram	BisGMA,UDMA,TEGDMA,EthoxylatedBis-EMA ,Barium,aluminium,silicate glass,ytterbium trifluoride, mixed oxide, Prepolymer	0.16–0.7 μm	80-81% by weight	Ivoclar Vivadent, Schaan, Liechtenstein	Z056PD
Tetric-N-bond universal	BisGMA (25-50%), water and ethanol (10-<25%), 2-hydroxyethyl methacrylate (HEMA) (10-<25%), phosphonic acid methacrylate (MDP) (10-<25%), diphenyl(2,4,6-trimethylbenzoyl) phosphine oxide (1-<2.5%), urethane dimethacrylate (0.3-<10)	N/A	N/A	Ivoclar Vivadent, Schaan, Liechtenstein	Z03RNR
N-Etch	37 % phosphoric acid in water, thickening agent, color pigments	N/A	N/A	Ivoclar Vivadent, Schaan, Liechtenstein	Z0370X
Admira fusion	Ormocer-aromatic, Barium aluminum	0.02-1 μm	84% by weight	Voco, Cuxhaven Germany	2335179
Admira Bond	Acetone, ormocer-based matrix, DMA, polyfunctional methacrylates, Ca stabilizer	N/A	N/A	Voco, Cuxhaven Germany	1809258
Vococid Etchant	35% orthophosphoric acid gel	N/A	N/A	Voco, Cuxhaven Germany	590722
Philips ZOOM!	25% HP,Water,potassium hydroxide, poloxomer 407, Eugenol, Propylene,Glycol, potassium nitrate,Mentha piperita,ferrous gluconate,glycerin, hydrogen peroxide	N/A	N/A	Discus,Dental,LLC Ontario CA91761, USA	19303025
Opalescence BOOST	40% HP, 20% water, 1.1% sodium fluoride and 3% potassium nitrate, potassium hydroxide.	N/A	N/A	Ultradent USA	BRKZP

**Table 2:** Comparison of roughness between types of composite and bleaching techniques.

		Control	Light	Chemical	ANOVA p-value	Control-light	Control-chemical	Light- chemical
Admira	X	.2127	.2411	.2510	.027*	.019*	.018*	.259
	SD	.0546	.0342	.0592				
Tetric	X	.2155	.2386	.2468	.024*	.017*	.047*	.338
	SD	.0460	.0307	.0363			
Independent samples t-test p-value		.891	.201	.420				

X; mean, SD; standard deviation; *p is significant at 5% level.

**Table 3:** Comparison of hardness between types of composite and bleaching techniques.

		Control	Light	Chemical	ANOVA p-value	Control-light	Control-chemical	Light- chemical
Admira	X	72.64	70.45	69.77	.049*	.047*	.027*	.696
	SD	3.12	3.19	2.05				
Tetric	X	76.08	73.83	73.03	.045*	.048*	.019*	.528
	SD	3.07	3.22	2.00				
Independent samples t-test p-value		.009*	.010*	.013*				

X; mean, SD; standard deviation; *p is significant at 5% level.

## Data Availability

The data that support the findings of this study are available from the corresponding author upon reasonable request.
